# Long-term day-by-day tracking of microvascular networks sprouting in fibrin gels: From detailed morphological analyses to general growth rules

**DOI:** 10.1063/5.0180703

**Published:** 2024-02-06

**Authors:** Katarzyna O. Rojek, Antoni Wrzos, Stanisław Żukowski, Michał Bogdan, Maciej Lisicki, Piotr Szymczak, Jan Guzowski

**Affiliations:** 1 Institute of Physical Chemistry, Polish Academy of Sciences, Warsaw, Poland; 2 Institute of Theoretical Physics, Faculty of Physics, University of Warsaw, Warsaw, Poland; 3 Laboratoire Matière et Systèmes Complexes (MSC), UMR 7057, CNRS & Université Paris Cité, Paris, France

## Abstract

Understanding and controlling of the evolution of sprouting vascular networks remains one of the basic challenges in tissue engineering. Previous studies on the vascularization dynamics have typically focused only on the phase of intense growth and often lacked spatial control over the initial cell arrangement. Here, we perform long-term day-by-day analysis of tens of isolated microvasculatures sprouting from endothelial cell-coated spherical beads embedded in an external fibrin gel. We systematically study the topological evolution of the sprouting networks over their whole lifespan, i.e., for at least 14 days. We develop a custom image analysis toolkit and quantify (i) the overall length and area of the sprouts, (ii) the distributions of segment lengths and branching angles, and (iii) the average number of branch generations—a measure of network complexity. We show that higher concentrations of vascular endothelial growth factor (VEGF) lead to earlier sprouting and more branched networks, yet without significantly affecting the speed of growth of individual sprouts. We find that the mean branching angle is weakly dependent on VEGF and typically in the range of 60°–75°, suggesting that, by comparison with the available diffusion-limited growth models, the bifurcating tips tend to follow local VEGF gradients. At high VEGF concentrations, we observe exponential distributions of segment lengths, which signify purely stochastic branching. Our results—due to their high statistical relevance—may serve as a benchmark for predictive models, while our new image analysis toolkit, offering unique features and high speed of operation, could be exploited in future angiogenic drug tests.

## INTRODUCTION

Microvascular tissue engineering is an emerging subfield in tissue engineering that focuses on the development of living capillary networks^1–3^ and vascularized microtissues for applications in drug testing,^4–9^ regenerative medicine,^10–13^ and general biofabrication.^3^ It is known that most types of engineered tissue constructs, if deprived of microvasculature while exceeding in size the so-called diffusion limit (appr. 1 mm), eventually suffer from hypoxia.^14^ Accordingly, the engineering of viable tissues at all scales necessarily requires the incorporation of an embedded microvasculature. One of the promising strategies toward efficient vascularization is angiogenesis, which is outgrowing or “sprouting” of new capillaries from preexisting vessels^15^ or from an endothelial monolayer,^6^ resulting in the formation of a branched hierarchical microvasculature. The sprouting vascular networks evolve via gradual elongation and bifurcation of the emerging sprouts. Despite the available large body of literature regarding the evolution of sprouting microvascular networks observed both *in vivo*,^16–18^ *ex vivo*,^17^ and *in vitro*,^6,17^ the dynamics of the branching phenomena in terms of topology of the evolving network, e.g., time-dependent changes in the number of branch generations or the distributions of branch lengths and branching angles, have not been studied experimentally in great detail. In particular, previous studies have mostly focused on providing morphological or dynamical characteristics, however, typically only at a single chosen time-point^19^ or at most at 2–4 distant time-points (e.g., days 1, 7, 14).^5,20–27^ In best cases, the growth has been monitored continuously (at every 1–2 h) for 4–6 consecutive days.^6,15,28^ Such time ranges were sufficient to estimate the overall trends in growth but too short to cover all stages of the typical microvascular evolution *in vitro*—that is from the emergence of sprouts to the saturation of their growth—which typically span altogether at least 14 days^22,25^ or even 21 days in some cases.^20,27^

Furthermore, sprouting microvasculatures have been frequently studied not in isolation but rather as a population of multiple microvasculatures dispersed randomly in the external extracellular matrix (ECM) and inosculating or otherwise biochemically interacting with each other.^15,26–28^ In fact, recent experiments with two neighboring vascular seeds^29^ demonstrated significant impact of such interactions on the final vascular morphology. Therefore, systematic studies on the dynamics of vascular sprouting should optimally rely on more controlled, reproducible initial conditions such as provided by a single isolated seed rather than multiple interacting seeds.

Here, we address all of the above issues by tracking the evolution of dozens of *isolated* sprouting microvascular networks day-by-day for *at least 14 days*, all cultured under similar, well-controlled conditions. We use the well-established bead-sprouting assay^30^ based on human umbilical cord endothelial cells (HUVECs) in which the microcapillary network sprouts from a HUVEC-coated microbead suspended in an external 3D hydrogel matrix (fibrin in our case). We focus on the case of HUVECs as an easily accessible and widespread source of endothelial cells, which are also frequently employed in angiogenesis assays.^31^ We develop a custom image analysis toolkit that facilitates the automated measurement of a variety of morphometric parameters including not only global characteristics, such as the overall length and area of the sprouts, but also statistical distributions of several observables, such as segment lengths, and branching angles. Our software, written in Python programming language, provides a solid background in terms of implementation and offers fast computation time. Overall, it is optimized for the processing of a large amount of data from multiple experiments. The time-resolved data, averaged over multiple experimental runs and spanning the whole lifetime of the networks, allow us to propose basic rules governing the topological development of the sprouting microvasculatures.

Quite surprisingly, even in the case of a single isolated bead, the details of the evolutionary dynamics remain poorly documented. In fact, to the best of our knowledge, the previous angiogenic bead-sprouting assays have been devoted mostly to the investigation of biological complexity of the sprouts, e.g., differentiation of the stalk and tip cells,^19,32–34^ the analysis of the simple network-morphometric parameters, such as the total length of the sprouts, total area, etc., at several time-points,^20,25,30^ or to the monitoring of tip-dynamics at short times (several hours up to 24 h).^35^ The problem of angiogenic dynamics has been previously addressed via theoretical means at various levels of complexity,^36–39^ including the case of a sprouting EC-spheroid.^37,40^ However, thus far, a direct comparison with the experiment has been limited due to the scarcity of the time-resolved experimental data and typically relied only on a qualitative comparison of late-time morphologies.^40^

Here, we focus on providing a solid experimental foundation for establishing a set of possibly general dynamic rules governing the evolution of the sprouting capillary networks. To this end, we carefully analyze the emerging microvascular topologies in terms of (i) *global observables*, such as the overall length and area of the sprouts, (ii) *microscopic observables*, such as branch lengths, branching angles (and their distributions), number of primary branches and positions of the tips of the sprouts, as well as (iii) *general measures of network complexity* such as the average number of branch generations. We also study the impact of the presence of fibroblasts in the ECM (either as a monolayer on top of the ECM or intermixed within the ECM) and the concentration of vascular endothelial growth factor (VEGF) in the culture media on the vascular growth dynamics. Shortly, we find sigmoidal growth patterns in which the onset of growth, yet not the speed of growth of individual sprouts, depends on the VEGF concentration. We also find that the distribution of the segment lengths is exponential, which provides evidence that the branching process is purely stochastic, whereas the bifurcation angle is typically in the range of 60°–75°, suggesting that, by comparison with the available diffusion-limited growth models, the bifurcating tips tend to follow local VEGF gradients.

Our methodology, based on the newly developed software, besides providing basic insight into the sprouting dynamics, also expedites the complex angiogenic image-analysis workflow and thus improves the standardization of the angiogenesis assays,^31^ which remains of significant relevance in a variety of drug tests and IC50 measurements.^9,41–44^ In further perspective, our results could also be used to guide the design of mesoscale vascular networks based on multiple interacting seeds, an emerging strategy in tissue engineering.^19,22^

## RESULTS

### Long-term time-lapse imaging of endothelial sprouting of single EC-coated beads

**FIG. 1.**
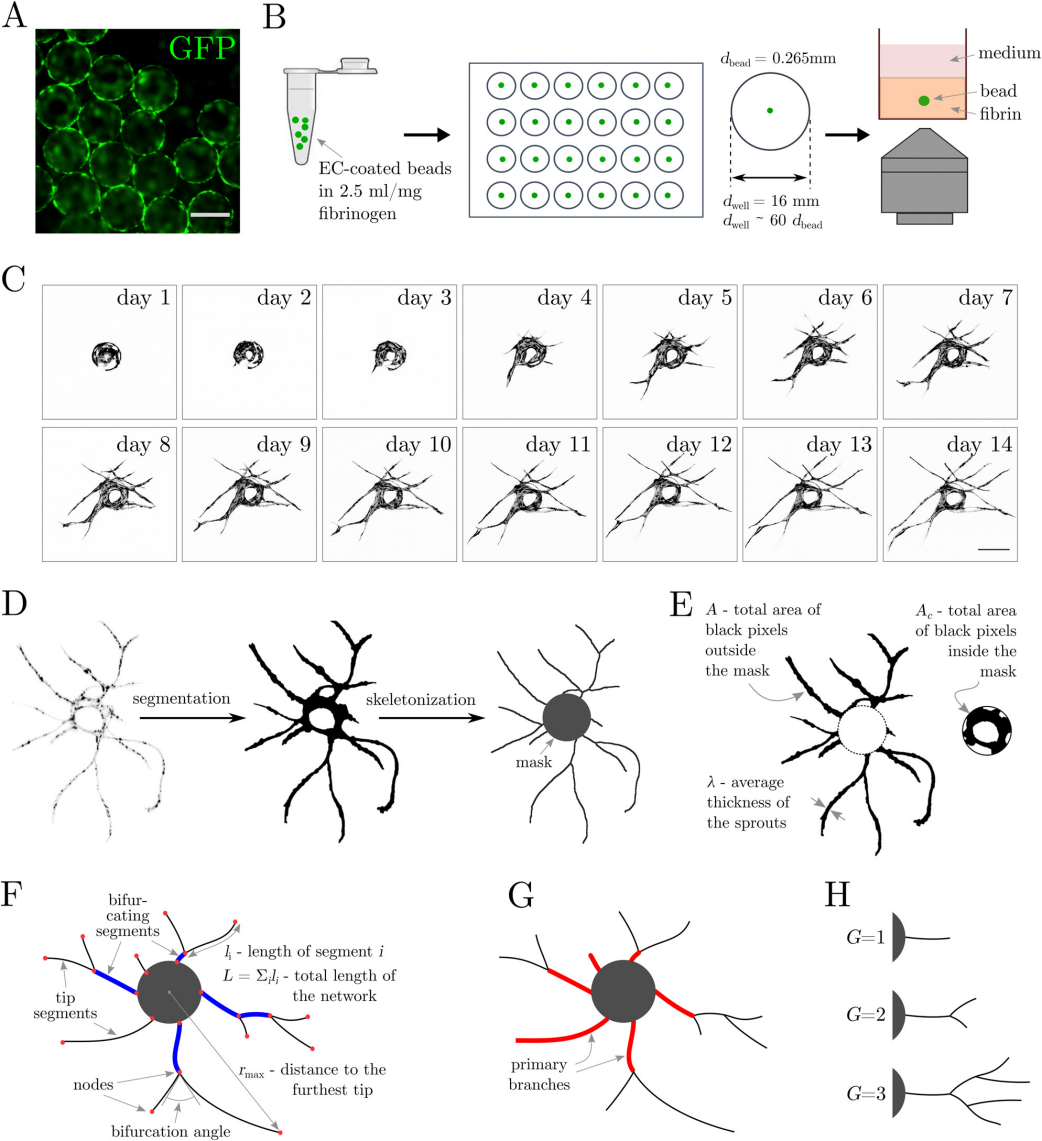
Long-term time-lapse imaging of the endothelial sprouting of single EC-coated beads: experimental design and image analysis workflow. (A) Polystyrene beads coated with GFP-transduced HUVECs. (B) Experimental workflow. Cell-coated beads are resuspended in fibrin solution, seeded one bead per well in a 24-well plate and imaged using confocal microscopy. (C) Representative confocal microscopy images of a single HUVEC-coated bead acquired at one day interval for 14 consecutive days. (D) Image processing workflow. From left to right: segmentation, skeletonization, and conversion to a graph. (E)–(H) Schematic representation of metrics used to characterize the networks including: (E) Area of the microvascular network *A* and the mask *A*_c_, and sprout thickness *λ*. (F) Length of the microvascular network *L*, its maximum span *r*_max_, bifurcation angles, bifurcating (blue) and tip (black) segments, and nodes (red). (G) Primary branches (shown in red). (h) Number of generations *G*. The scale bars in [(A), (C)] are 250 *μ*m.

To study the morphogenesis of vascular networks, we examined the dynamics of EC sprouting from isolated EC-coated beads during the *in vitro* angiogenic process. We coated polystyrene microbeads with green fluorescent protein (GFP)-tagged human umbilical vein endothelial cells (HUVECs) [Fig. 1(A)] and embedded them in 2.5 mg/ml of fibrin hydrogel. The beads were seeded in 24-well plates, with one bead per well [Fig. 1(B)] to exclude the potential impact of the presence of nearby beads on the directionality and/or growth dynamics of the angiogenic sprouts.^29^ Also, to provide possibly isotropic initial conditions, the beads were positioned centrally in the wells, at a large well-to-bead size ratio *d*_well_/*d*_bead_ ≈ 60. The thickness of the hydrogel layer was approximately 1.5 mm. During the assay, we observed several stages of the angiogenic process starting with the formation of an endothelial layer at the surface of the bead, followed by sprouting of ECs from the layer, sprout elongation, bifurcation, and finally the formation of a characteristic dendritic star-like architecture. To characterize the evolution of the global properties of the network over time, we visualized the morphology of the angiogenic sprouts around each of the EC-coated beads using confocal microscopy. We acquired the images at 24 h time intervals over a period of 14 days to cover all stages of the angiogenic morphogenesis [Fig. 1(C)]. We placed the samples under a confocal microscope once per day for a short time to acquire an image of the EC-coated bead, whereas in between the imaging sessions, the sample was placed in the CO_2_ incubator. Such intermittent imaging sessions limited the time of exposure of the cells to non-physiological conditions outside the CO_2_ incubator, supported cell viability, and extended their lifetime. We observed cells to remain viable for at least 14 days and even up to 20 days in some cases (supplementary material, Fig. 1).

The proposed approach allowed us to track the evolution of a high number of sprouting networks in parallel. From a practical point of view, the setup did not require the supply of any additional medium; the medium could be easily changed under a laminar hood between imaging sessions. Our day-by-day analysis goes beyond the previous studies, which typically focused either on a single culture time-point, e.g., day 7 or day 14 (Refs. 20, 25, and 30) or studied the EC sprout outgrowth during a relatively short window of time, usually limited to 1–2 days at high image acquisition frequency.^17,32^

### Image analysis

To efficiently analyze the large amount of generated data, we have developed an automated image processing tool. The software overlays confocal microscopy images of a given sample acquired at multiple time-points to allow the tracking of sprout evolution around each given bead. The program extracts the morphology of a sprouting network directly from the experimental image and produces a database of the measured metrics (bifurcation and termination points, segment lengths, branching angles, etc.), which are further analyzed statistically [Figs. 1(D)–1(H)]. We use Python as the programming language, which offers open-source image and data processing libraries and significantly simplifies the code.^45–52^

Our image processing workflow [Fig. 1(D)] starts with image segmentation. The software detects the EC-coated polystyrene bead, defines a corresponding circular “mask,” and identifies the sprouts. Next, it calculates the projected area of the cells inside the mask (*A*_c_), i.e., the area of the cells covering the bead, and outside the mask (*A*), i.e., the total area of the sprouts [Fig. 1(E)]. Then, it performs the skeletonization of the previously segmented image and saves it as a custom graph class [Fig. 1(F)]. At this stage, based on the detected skeleton, the software calculates the total length of the vascular network (*L*), and the mean width of the sprouts (*λ* = *A*/*L*); see Figs. 1(E) and 1(F). Nodes of the graph [Fig. 1(F)] are identified as one of the following: (i) a branching point, (ii) a base of a sprout (the point of contact with the mask), or (iii) a sprout tip. The sprout *segments* are accordingly identified as the parts of sprouts contained between two neighboring nodes. In particular, the software separately classifies *tip segments*, that is, the segments terminating with a sprout tip, i.e., non-bifurcating ones, and *bifurcating segments*. In the following, we use subscripts *tip* or *bif* to distinguish between the two types of segments.

Next, the program calculates radial coordinates of the tips and finds their maximum spread (*r*_max_). It also finds the number of tips (*N*_tip_) and the number of primary branches (*N*_pb_), i.e., segments in direct contact with the central bead [Fig. 1(G)]. The complexity of the vascular network is assessed by computing the average number of generations *G* = 1 + log_2_(*N*_tip_/*N*_pb_) originating from primary branches.^53^ Note that in the case of a simple tree-like network originating from a single branch (*N*_pb_ = 1), we have *G* = 1 + log_2_(*N*_tip_); see Fig. 1(H).

Finally, our code measures the bifurcation angles *ϕ* [Fig. 1(F), supplementary material, Fig. 5(a)]. To this end, in particular, the software applies a filter, which allows one to distinguish (in most cases) between bifurcations and anastomosis events and focuses only on the former. We measure the branching angle in order to allow comparison with the available morphogenesis models.

### The interstitial distribution of fibroblasts promotes endothelial sprouting of EC-coated beads

**FIG. 2.**
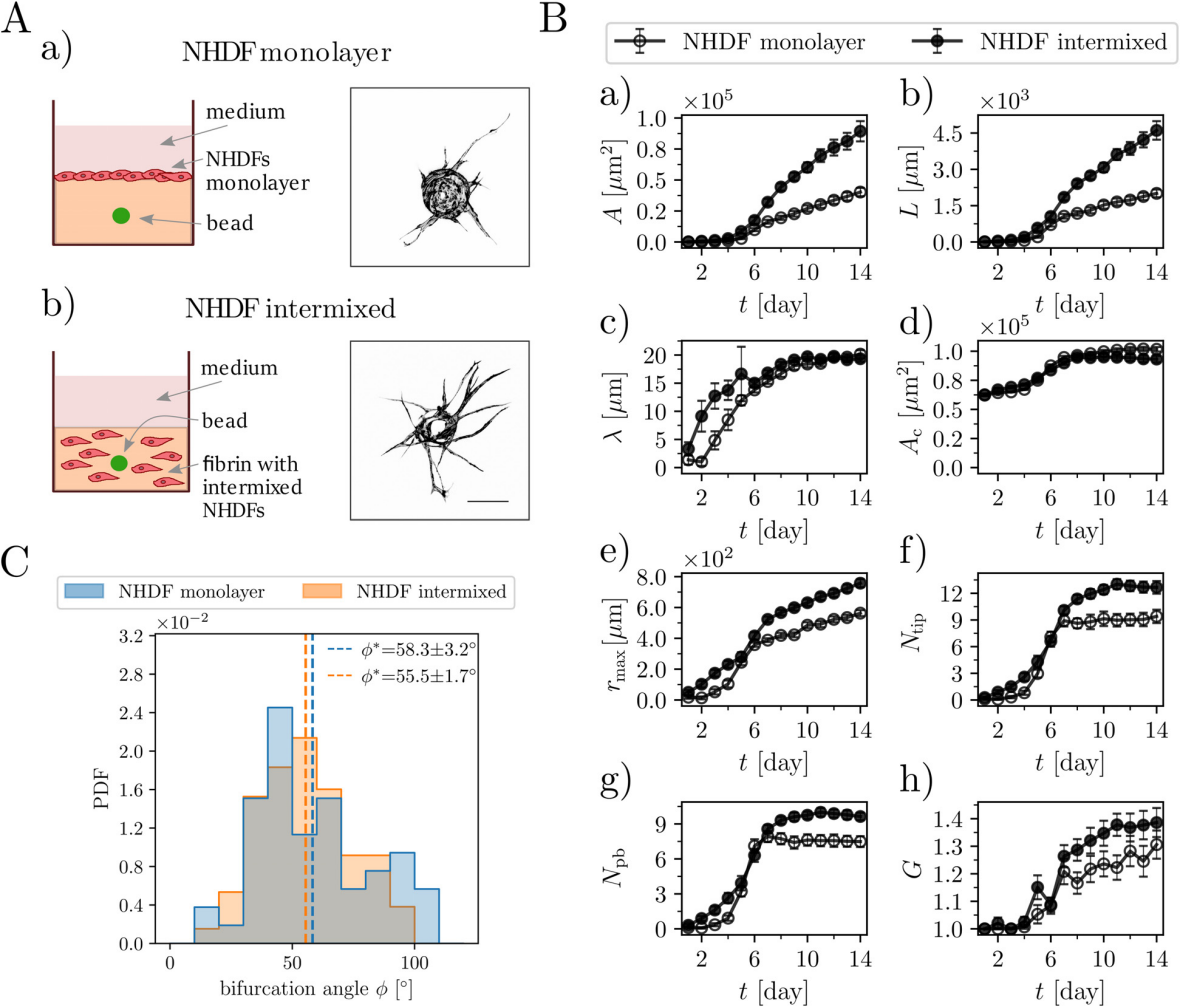
Distributing NHDFs through the matrix promotes the endothelial sprouting of EC-coated beads. (A) Different seeding conditions of NHDFs during the angiogenesis bead-sprouting assay—schematic drawings and representative images of single GFP-tagged HUVEC-coated beads. NHDFs were either seeded as (a) a monolayer on the top of a fibrin clot or (b) distributed throughout the fibrin matrix. Day 10 of culture. Scale bar 250 *μ*m. (B) Morphometric analysis of (a) the total area *A*, (b) the total length *L*, (c) the average sprout width *λ,* (d) the area of the cell-coated bead *A_c_*, (e) the distance to the furthest tip *r*_max_, (f) the number of tips *N_tip_*, (g) the number of primary branches *N_pb_*, and (h) the average number of generations *G* per branch [*G =* 1+log_2_(*N_tip_/N_pb_*)] of the sprouting capillary networks for 14 consecutive days. The numbers of beads in the assay (biological repetitions) were *n* = 37 in the NHDF-monolayer case and *n* = 36 in the NHDF-intermixed case. The error bars correspond to the standard error of the mean (SEM). (C) Distribution bifurcation angles for the monolayer and intermixed fibroblast seeding conditions. The presented values are the mean ± SEM. NHDF-monolayer, *n* = 53; NHDF-intermixed, *n* = 131; *p* > 0.05. The overlap between the histograms is rendered in gray. “PDF” refers to the probability density function. Statistical significance was analyzed using the Shapiro–Wilk test and the U Mann–Whitney test (C) and two-way analysis of variance followed by Bonferroni's test (B). Numerical values that underlie the graphs are shown in S1 Data. See S1 Table for detailed statistics for (B).

It is known that fibroblasts act as supporting cells and surround capillary-like structures, promoting the formation of stable vascular networks.^54^ The fibrinolytic activity of fibroblasts leads to a remodeling and the gradual degradation of the fibrin matrix.^54^ This enhances the diffusive transport of fibroblast-derived proangiogenic factors, such as the vascular endothelial growth factor (VEGF), angiopoietin-1, and platelet-derived growth factor (PDGF), and, in general, has a supportive effect on the diffusion of the cell culture media throughout the fibrin matrix.^20^ In the first series of experiments, we aimed at examining the impact of the distribution of normal human dermal fibroblasts (NHDFs) within the fibrin matrix on the morphogenesis of the vascular network around an isolated EC-coated bead. NHDFs were either distributed in the bulk of the fibrin gel (the “intermixed” case) or seeded at the gel-media interface forming a cellular monolayer (the “monolayer” case); see Fig. 2(A) and supplementary material, Fig. 2. We observed that, in qualitative agreement with previous studies,^20,25,54^ distributing fibroblasts interstitially promoted sprouting and led to a faster network development as compared to the case of a monolayer. This was reflected in a significant increase in the total area *A* and the length *L* of the vascular networks at all times; see Figs. 2(B-a) and 2(B-b). The difference was already apparent at day 5 of culture and gradually increased, reaching the maximum at day 14. Also, starting from day 7, the rate of growth of both *A* and *L* appeared approximately three times higher in the “intermixed” case, with the measured values doubling those observed in the “monolayer” case at day 14. The observed dynamics strongly correlated with the dynamics of the number of primary branches *N*_pb_, the number of tips *N*_tip,_ and the distance to the furthest tip *r*_max_ [Figs. 2(B-e)–2(B-g)], despite somewhat smaller differences between the “intermixed” and the “monolayer” configurations in these cases. Overall, in the “monolayer” case, the dynamics of *A*, *L*, and *N*_pb_, *N*_tip_, *r*_max_ significantly slowed down, starting from day 6 or 7 (depending on the observable), whereas in the “intermixed” case, the pace of growth remained high until much later times, that is, day 14 (for *A* and *L*) or day 11 (for *N*_pb_ and *N*_tip_). Considering the maximal distances *r*_max_ span by the networks, the “intermixed” networks grew wider by roughly 200 *μ*m as compared to the “monolayer” networks. Interestingly, the difference emerged in a stepwise manner, with the jump occurring around days 7 and 8, and remained almost unchanged at later times. Hence, it seems that distributing fibroblasts inside the ECM tends to extend the period of intense sprout elongation. The complexity of the network, as measured by the number of branch generations *G*, remained slightly elevated in the “intermixed” case, starting from day 8 [Fig. 2(B-h)]. This suggests that distributing NHDFs through the fibrin matrix leads to the formation of bigger (in terms of *L*, *A*) and slightly more branched (*G*) vascular networks. Finally, the number of cells covering the bead, as measured by *A*_c,_ appeared not to be significantly affected by the fibroblast distribution [Fig. 2(B-d)]. The values measured in the “monolayer” case remained around 10% higher than in the “intermixed” case. The shortage of bead-coating cells in the “intermixed” case could be related to the higher number of cells migrating from the bead toward the sprouts in this case. The average width of the sprouts (*λ*) was very similar in both cases, starting from day 6, i.e., once the sprouting set off in both cases [Fig. 2(B-c)].

### The impact of fibroblast distribution on bifurcation angles

As branching events are crucial for the evolving topology of the sprouting tree-like networks, here we analyze in more detail the distributions of the branching angles. The angles between branches have been measured previously during neovascularization *in vitro*.^28,55^ However, in those cases, the direction of tip growth was not identified, so that the measurements have not distinguished between the branching and the anastomosis events. Here, we exploit the radial growth of the sprouts and apply a corresponding exclusion rule in the algorithm (see Methods), which allows us to filter out most of the anastomosis events and to perform more precise branching angle measurements.

Figure 2(C) shows the histograms of bifurcation angles (*ϕ*) for the advanced stage of vascular network growth (day 12 of culture) for both “intermixed” and “monolayer” cases. We observe quasi-Gaussian distributions (verified for normality using a Shapiro–Wilk test, see S1 Table) centered around the mean values of ϕ^*^ = 55.5 ± 1.7° and ϕ^*^ = 58.3 ± 3.2°, respectively, with the error estimated as the standard error of the mean. Accordingly, we may conclude that the type of spatial fibroblast distribution has little effect on the observed branching angle, as suggested by the results from a U Mann–Whitney test, used for comparing non-Gaussian distributions; see S1 Table. However, in the “monolayer” case, the total number of bifurcation events pooled from all experiments is not excessive, *n* = 53, and it is difficult to draw strong conclusions regarding the measured distribution or the mean value. As we show below, the situation is improved as the size and complexity of the networks increase, which we achieve through increasing the concentration of VEGF in the culture media. At high VEGF concentrations, the number of bifurcation events exceeds *n* = 190, which significantly raises the statistical relevance of the results.

### The concentration of VEGF-A-165 determines the onset of angiogenic sprouting, growth dynamics, and final morphology of microvessels

**FIG. 3.**
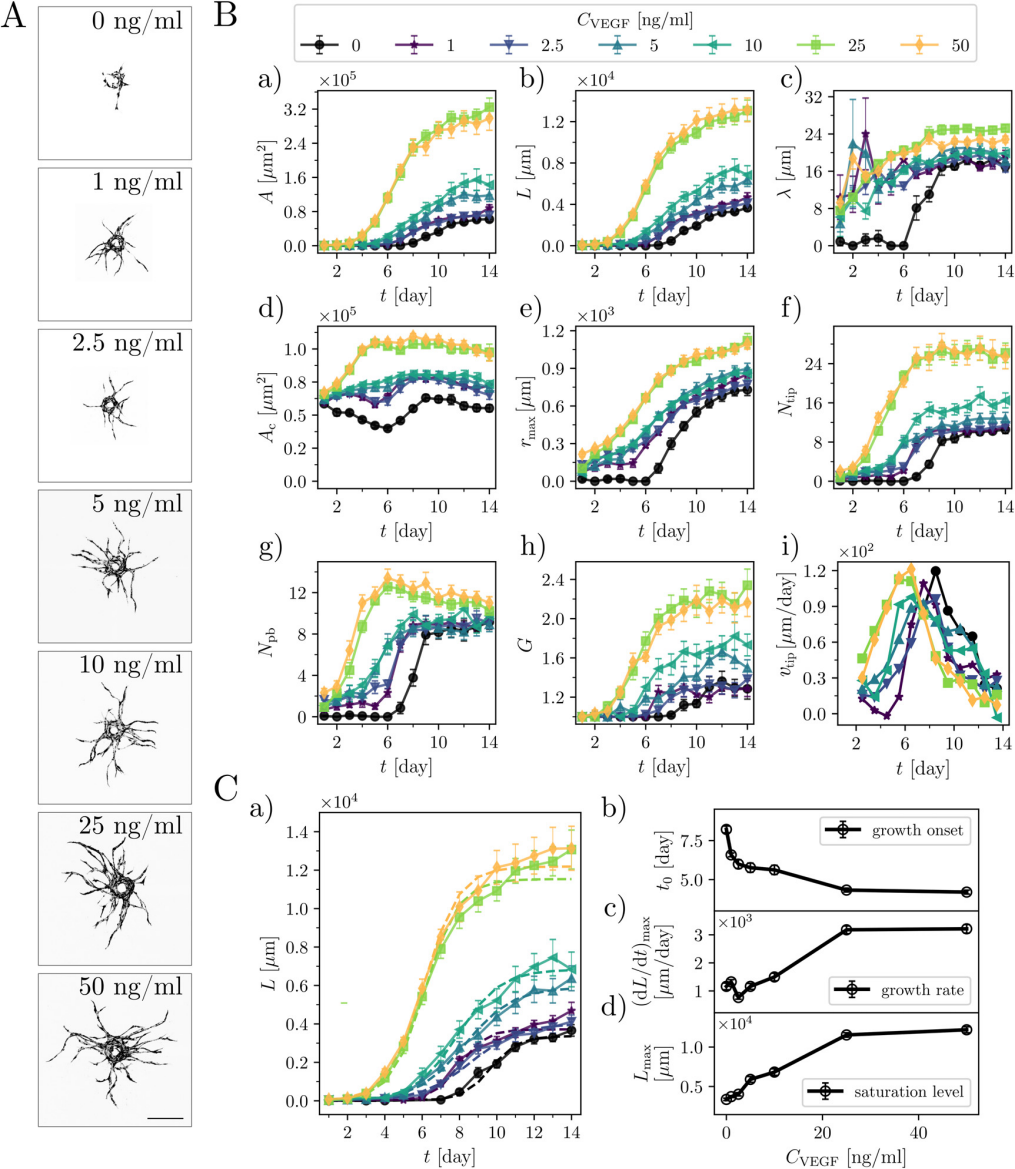
VEGF-A concentration determines the onset of angiogenic sprouting, the rate of growth, and the final morphology of the capillary networks. (A) Representative images of GFP-tagged HUVEC-coated beads cultured at the indicated VEGF-A concentrations, day 10. Scale bar 250 *μ*m. (B) Morphometric analysis of sprouting capillary networks at the indicated VEGF concentrations in terms of (a) the total area *A*, (b) the total length *L*, (c) the average sprout width *λ* (d) the area of the cells coating the bead *A_c_*, (e) the distance to the furthest tip *r*_max_, (f) the number of tips *N_tip_*, (g) the number of primary branches *N_pb_*, (h) the average number of generations *G* per branch [*G* = 1 + log_2_(*N*_tip_/*N_pb_*)], and (i) the average tip velocity *v*_tip_ (the plot shows the moving average with the averaging time of 2 days). The numbers of beads (biological repetitions) taken for the statistics were as follows: *C*_VEGF_ = 0 ng/ml, *n* = 13; *C*_VEGF_ = 1 ng/ml, *n* = 13; *C*_VEGF_ = 2.5 ng/ml, *n* = 14; *C*_VEGF_ = 5 ng/ml, *n* = 14; *C*_VEGF_ = 10 ng/ml, *n* = 12; *C*_VEGF_ = 25 ng/ml, *n* = 14; *C*_VEGF_ = 50 ng/ml, *n* = 12. Symbols in the graphs indicate the mean values, and error bars are the standard error of the mean (SEM). (C) The parameters describing the growth process. (a) Comparison between the experimental data *L*(*t*) and fitted curves (a logistic function), see Eq. (1), for various *C*_VEGF_. (b) The VEGF-dependence of the onset of growth *t*_0_, (c) the rate of growth (d*L*/d*t*)_max_, and (d) the saturation level *L*_max_. In (a), the error bars correspond to the SEM, whereas in the remaining panels to the standard errors of the parameters obtained using the least squares method. Statistical significance was analyzed using a two-way analysis of variance followed by Bonferroni's test (B). Numerical values that underlie the graphs are shown in S1 Data. See S1 Table for detailed statistics for (B). For (C), the accuracy of the fit was evaluated using two metrics: mean absolute percentage error (MAPE) and weighted mean absolute percentage error (WMAPE); see S1 Table for numerical values and Material and Methods section for an explanation of the metrics.

Vascular endothelial growth factors (VEGFs) are essential for the induction of angiogenesis and drive both EC proliferation and migration.^56,57^ The VEGF family consists of five members: VEGF-A, VEGF-B, VEGF-C, VEGF-D, and placental growth factor (PLGF). VEGF-A isoform is the one most abundant in humans^58,59^ and has emerged as the single most important regulator of the blood vessel formation in health and diseases. It is essential for embryonic vasculogenesis and angiogenesis and is a key mediator of neovascularization in cancer and other diseases.^60^ To characterize the impact of VEGF on EC sprouting dynamics, we added increasing concentrations (*C*_VEGF_) of human recombinant VEGF-A-165 into the fibrin bead-sprouting assay and quantified the global morphometric parameters of the vascular networks formed. We performed a dose–response titration for a series of VEGF-A-165 concentrations *C*_VEGF_ = [0, 1, 2.5, 5, 10, 25, 50] ng/ml in the medium added to each well in the presence of NHDFs interstitially distributed within the fibrin matrix (the “intermixed” configuration). As in previous experiments (without the added VEGF), here, we also visualized isolated EC-coated beads at 24 h time intervals over the period of 2 weeks. We observed that, as expected from the previous literature,^30,61–63^ the concentration of VEGF had a significant impact on the EC sprouting dynamics and overall complexity of the ensuing microvascular networks [Fig. 3(A) and supplementary material, Fig. 3]. The EC-coated beads treated with a higher *C*_VEGF_ formed larger and more branched vascular networks and started to sprout earlier [Fig. 3(B)]. Increasing *C*_VEGF_ from 0 ng/ml to 25 ng/ml led to an approximately sixfold increase in the final network area *A* and a three- to fourfold increase in the final total length *L* [Figs. 3(B-a) and 3(B-b)]. Accordingly, we also observed a 1.5 to twofold increase in the final thickness of the branches, *λ* [Fig. 3(B-c)]. Increasing *C*_VEGF_ further up to 50 ng/ml did not result in further changes, suggesting a saturation of the system at around 25 ng/ml. The observed changes of the area of the mask, *A*_c_ [Fig. 3(B-d)], indicate that the VEGF concentration affected not only the formation of sprouts but overall had a significant impact on the HUVEC proliferation during the assay. In particular, higher concentrations of VEGF resulted in an increased final area of the mask *A*_c_, indicating a more frequent cell proliferation at the monolayer, whereas the lack of the exogenous VEGF (the case 0 ng/ml) led to a decrease in *A*_c_ in the initial stage of culture. The latter observation could be explained in terms of the arrest of HUVEC growth and/or their apoptosis caused by an insufficient supply of VEGF, resulting in a regression of the vascular network.^64–66^

The maximum distance *r*_max_ spanned by the networks, the numbers of tips *N*_tip,_ and the primary branches *N*_pb_ were also significantly elevated for cultures treated with a higher *C*_VEGF_ [Figs. 3(B-e)–Fig. 3(B-g)]. Vascular networks exposed to higher VEGF concentrations sprouted earlier and, in general, developed a more complex topology, as reflected by the higher final number of branch generations, *G* = 1 + log_2_(*N*_tip_/*N*_pb_) [Fig. 3(B-h)]. The increase in *G* was particularly pronounced for cultures treated with *C*_VEGF_ = 25 and 50 ng/ml. This increase in complexity could be attributed, in general, either to the earlier onset of sprouting or to the possible faster linear growth of the sprouts. To verify the latter possibility, we determined the ensemble-averaged linear speed of the tips. We used the formula *v*_tip_ = Δ*L*/(Δ*t N*_tip_), where Δ*t* is the interval between the measurements, that is, one day, and Δ*L* is the corresponding increase in the net length *L* of the network (i.e., we assumed that the network grows only at the tips). We found that, independently of the VEGF concentration, the velocity *v*_tip_ as a function of time always developed a maximum shortly after the onset of growth [Fig. 3(B-i)]. Importantly, we also observed that the corresponding maximal velocities were of similar magnitude, independently of *C*_VEGF_. Accordingly, we may conclude that the presence of more evolved networks at higher *C*_VEGF_ is associated rather with the earlier onset of sprouting and thus with the overall longer period of growth (in each case extending until saturation at around day 11) rather than with the tip velocity *v*_tip_.

In the following, we turn to a more detailed analysis of the evolution of sprouting networks over time depending on *C*_VEGF_. First, we observe that the two main parameters describing the morphogenesis of a vascular network, i.e., *L*(*t*) and *A*(*t*), exhibit a sigmoidal growth pattern with an initial inactive phase (no sprouts) followed by an exponential growth phase and a final plateau phase. The growth curves *L*(*t*) and *A*(*t*) can be approximated each by a logistic curve of the form

$$Y(t)=Y_{\text{max}}/(1+\text{exp}(–\;k(t-t_{1}))),$$ (1)

where *Y*_max_ is the saturation level, *k* is the characteristic growth rate in the exponential phase, and *t*_1_ is the inflection point of the sigmoid corresponding to the moment of the fastest growth. From the fitted curves, we extract the maximal rate of growth (d*Y*/d*t*)_max_ = *Y*_max_*k*/4 and the onset of growth *t*_0_ = *t*_1_ − 2/*k* (which corresponds to the x-intercept of the tangent line to the graph Y(t) at the inflection point). The proposed fits seem to accurately describe the dynamics of EC sprouting [see Fig. 3(C) for the length *L* and supplementary material, Fig. 4 for the area *A*]. Some discrepancies (e.g., no obvious plateau phase in some cases) are observed during the final days of the experiment (approximately after day 10 of the assay), and they seem to be more pronounced for higher VEGF concentrations. This is possibly caused by the longer period of the extensive growth of the network in these cases, with the growth only starting to saturate at around day 10. Nevertheless, we have decided not to replace Eq. (1) with a more sophisticated formula that involves a larger number of parameters, as this could lead to overfitting and issues with interpretability. It is important to note that the estimation of the onset time and the maximal growth rate is not impacted by the long-term behavior of the curves. Therefore, fitting with Eq. (1) should yield accurate values for these parameters. We find that the onset of the sprout elongation *t*_0_ systematically decreases with *C*_VEGF,_ reaching a plateau of *t*_0_ = 4 [days] at concentrations *C*_VEGF_ = 25 and 50 ng/ml. This means that increasing *C*_VEGF_ in the range [0, 25] ng/ml expedites sprouting [Fig. 3(C-b)]. At low VEGF concentrations (*C*_VEGF_ = 2.5 ng/ml), the maximum growth rates of the sprout length (d*L*/d*t*)_max_ [Fig. 3(C-c)] and of the sprout area (d*A*/d*t*)_max_ [supplementary material, Fig. 4(A-c)] are nearly independent of *C*_VEGF_, whereas they are roughly proportional to *C*_VEGF_ in the regime 2.5 ng/ml < *C*_VEGF_ <25 ng/ml and saturate above *C*_VEGF_ = 25 ng/ml. Similar scenarios with a somewhat steadier increase already at the lower *C*_VEGF_ are also observed for the saturation levels *L*_max_ and *A*_max_ [Fig. 3(C-d) and supplementary material, Fig. 4(A-d)].

**FIG. 4.**
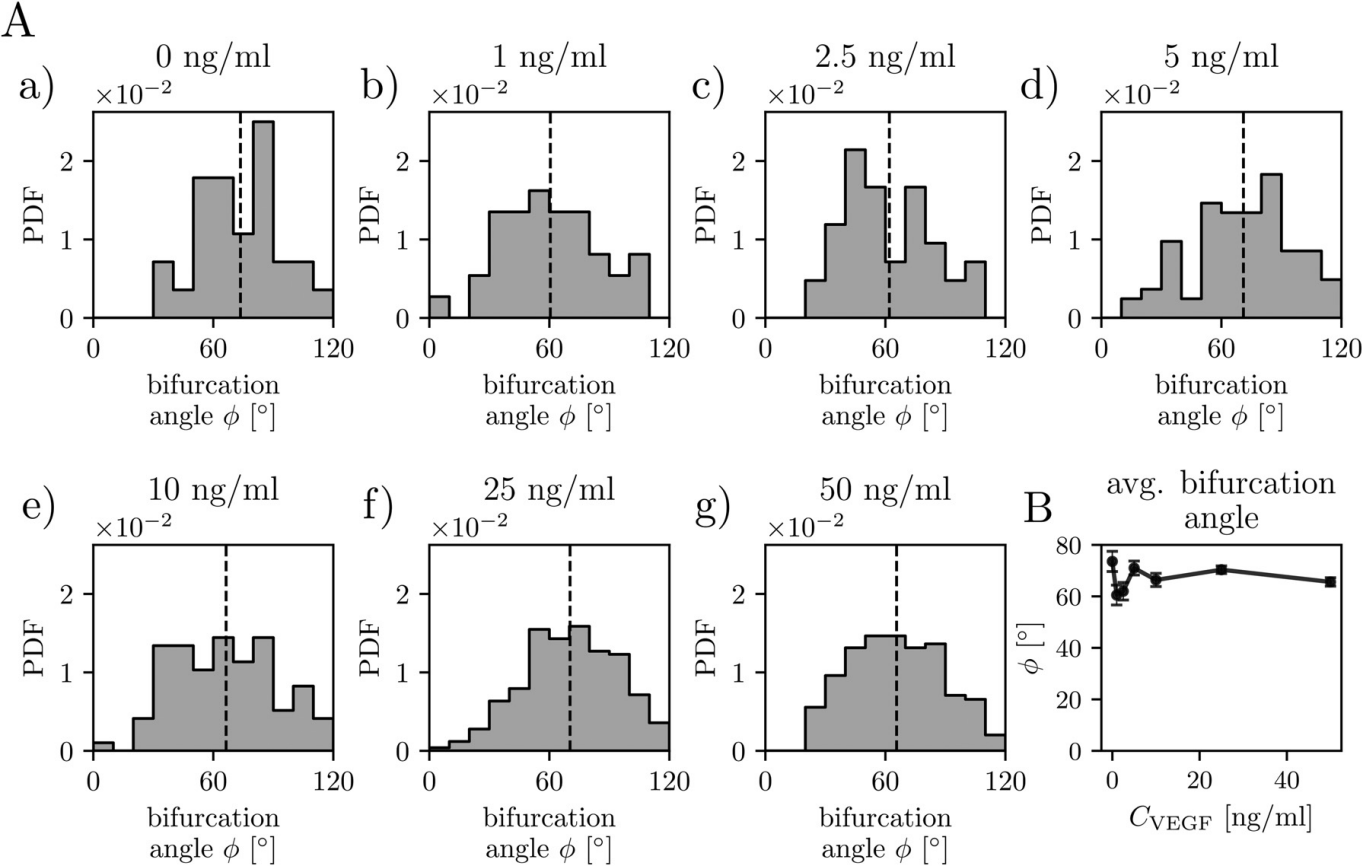
VEGF-A concentration does not affect the distribution of bifurcation angles. (A) Analysis of the bifurcation angle distributions for indicated VEGF concentrations at day 12 of culture. The total numbers *n* of the measured angles were (a) *C*_VEGF_ = 0 ng/ml, *n* = 28; (b) *C*_VEGF_ = 1 ng/ml, *n* = 37; (c) *C*_VEGF_ = 2.5 ng/ml, *n* = 42; (d) *C*_VEGF_ = 5 ng/ml, *n* = 82; (e) *C*_VEGF_ = 10 ng/ml, *n* = 97; (f) *C*_VEGF_ = 25 ng/ml, *n* = 252; and (g) *C*_VEGF_ = 50 ng/ml, *n* = 198. The dashed lines show mean values. “PDF”—probability density function. (B) The mean bifurcation angle plotted as a function of *C*_VEGF_. The error bars are the standard error of the mean. Statistical significance was analyzed using a one-way analysis of variance followed by Tukey's test (B). Numerical values that underlie the graphs are shown in S1 Data. See S1 Table for detailed statistics for A.

**FIG. 5.**
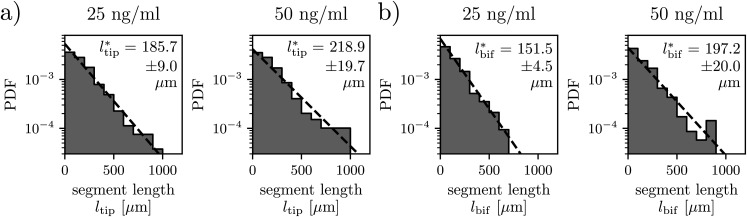
Capillary networks formed at high VEGF concentrations display the exponential segment length distributions. Analysis of the distribution of the length of (a) tip segments and (b) bifurcating segments for *C*_VEGF_ = 25 and 50 ng/ml. Numbers of identified segments were (a) *C*_VEGF_ = 25 ng/ml, *n* = 266; *C*_VEGF_ = 50 ng/ml, *n* = 198, (b) *C*_VEGF_ = 25 ng/ml, *n* = 426; *C*_VEGF_ = 50 ng/ml, *n* = 348. The characteristic segment lengths are indicated on the graphs with the standard errors. “PDF” refers to the probability density function. Numerical values that underlie the graphs are shown in S1 Data.

Finally, we analyze the impact of the VEGF concentration on the distribution of the bifurcation angle *ϕ* (Fig. 4) and the segment length *l* (Fig. 5). In the case of angle measurements, we first validate our methodology by comparing the results with a manual measurement of the bifurcation angles. Next, we tune the internal parameter of the angle-measurement algorithm (the “arm length;” for details, see Materials and Methods section, supplementary material, Fig. 5) to match the manual measurements best. With the optimized algorithm, we find that the average bifurcation angle ϕ^*^ varies in the range from 61° to 72° as *C*_VEGF_ increases from 0 to 50 ng/ml. We observe a shallow minimum at *C*_VEGF_ around 1–2 ng/ml. For higher *C*_VEGF_, ϕ^*^ stabilizes at around 65°–68°. In the case *C*_VEGF_ = 2.5 ng/ml, we observe two peaks in the bifurcation angle distribution, which might indicate a transition between the effects of small *C*_VEGF_ and the release of bound VEGF from the surrounding fibrin matrix, which becomes important at higher VEGF concentrations.^67^

Considering the distributions *P*(*l*) of the segment lengths *l*, we also verify the validity of the automated image analysis via comparing with manual image segmentation. In general, we find a reasonable agreement between the automated and the manual measurements without any tunable parameters (supplementary material, Fig. 6). The distributions differ only at very small *l*, where apparently the numerical approach overestimates the number of the shortest segments. The overpopulation of the short segments can be attributed to the skeletonization procedure, which tends to produce many “artificial” short segments in the regions of high sprout density.

Based on the results for all studied *C*_VEGF,_ we find that at low *C*_VEGF,_ the total number of segments is too low to draw statistically meaningful conclusions about the distribution (supplementary material, Fig.7). Therefore, we limit the more detailed analysis to only the highest VEGF concentrations, that is, the cases *C*_VEGF_ = 25 and 50 ng/ml. In these two cases, we observe strong evidence for the exponential decay of *P*(*l*) (Fig. 5 and supplementary material, Fig. 6; the latter one shows the results of manual measurements). The exponentially decaying distributions are observed for both tip segments *P*(*l*_tip_) as well as for the bifurcated segments *P*(*l*_bif_), which is verified via fitting *P*(*l*)∼exp(−*l*/l^*^), where l^*^ is the average length of a segment. Such a division is meaningful since the population of the actively growing segments (tip) may have different statistics than the population of segments that have completed growth (bif).

In general, the exponential probability distributions are characteristic of a Poisson process in which the events (bifurcations in this case) occur randomly in space, yet at a constant average spatial density, given by 1/l^*^. Another conclusion that can be drawn from Fig. 5 and supplementary material, Fig. 6, is that the average length of a bifurcated segment (l^*^_bif_) is, in general, close to the average length of a tip segment (l^*^_tip_). We observe a statistically significant difference only in the case of beads treated with *C*_VEGF_ = 25 ng/ml (S1 Table). In fact, this case provides particularly rich statistics with the highest total number of segments (e.g., *n* = 692 for *C*_VEGF_ = 25 ng/ml vs *n* = 546 for *C*_VEGF_ = 25 ng/ml) and the most branched networks [highest *G* at day 14, see Fig. 3(B-h)]. In this case, we find that l^*^_bif_ < l^*^_tip_ with the difference being statistically significant according to the 3-sigma test (see “Statistical analysis” in the Methods section). This observation could be explained by noting that, given sufficient time, an actively growing tip segment will eventually split and become a bifurcated segment; thus, the long “tail” in the distribution builds first in *l*_tip_ distribution and only later becomes visible in *l*_bif_. Finally, no statistically significant difference in either l^*^_bif_ or l^*^_tip_ between the cases with *C*_VEGF_ = 25 ng/ml and *C*_VEGF_ = 50 ng/ml is observed. We verify these conclusions by employing both algorithmic and manual measurements of the segment lengths and employing the 2-sigma and 3-sigma tests (see S1 Table). Noteworthily, we also find that the numerical artifact associated with the overpopulation of the shortest segments, which we systematically observe in the algorithmic measurements, has little effect on the fitted values l^*^_bif_ and l^*^_tip_ (supplementary material, Fig. 6).

## DISCUSSION

### Growth rules

In summary, according to our study, the angiogenic sprouts in the HUVEC bead-sprouting fibrin assays seem to obey the following rules of growth:

(1) The evolution of the sprouting networks proceeds via three stages: (i) inactive stage during which the cells proliferate only within the monolayer/multilayer covering the bead, (ii) exponential growth stage, consisting of rapid sprout elongation and branching, and (iii) maturation stage during which the growth slows down and eventually saturates.
(2) Distributing fibroblasts interstitially in the fibrin gel promotes HUVEC sprouting and leads to more branched capillary networks as compared to the case with fibroblasts growing on top of the fibrin clot. This confirms previous findings.^20^
(3) Increasing the concentration of the vascular endothelial growth factor (VEGF) in the media leads to earlier sprouting (via shortening of the initial inactive stage) and leads to an increased number of primary branches, yet without significantly affecting the linear speed of sprout growth.
(4) At larger VEGF concentrations (25–50 ng/ml), the distributions of segment lengths (i.e. distances between the bifurcation points) become exponential, a feature characteristic of a network subject to random uncorrelated branching, in mathematical statistics referred to as the Poisson process. Accordingly, our results support a picture of a stochastically branching network, in line with some of the available branching morphogenesis models, such as, e.g., the branching and annihilating random walk (BARW).^68–70^ Interestingly, the characteristic segment length (the inverse of the decay rate of the distribution) does not seem to depend on the VEGF concentration.
(5) The average values of the branching angle vary in the range of 60°–75°, i.e., close to (2π/5) × 180 = 72° characteristic of Laplacian growth models, i.e., diffusion-limited growth models,^71–74^ which overall suggests that the tips follow the local gradients of the VEGF concentration.

Collectively, our results, due to their high statistical relevance, may serve, e.g., as a benchmark for predictive models. Below, we discuss our findings in more detail.

### Fibroblast distribution

The role of fibroblast distribution in the matrix and the VEGF concentration in the assay have been a subject of intense studies in previous years. Currently, there is a consensus^20,25,54^ that fibroblasts distributed throughout the hydrogel matrix tend to soften the matrix and promote the diffusion of growth factors, which in turn promotes EC sprouting. Our study also confirms this scenario.

### Impact of VEGF concentration

The impact of VEGF, in particular, the VEGF concentration in the medium, on the morphology of a vascular network, including the topological network characteristics—due to the available contradicting experimental evidence—has remained an open issue.^30,44,62,75,76^ In the present study, we report that increasing the VEGF-A-165 concentration, *C*_VEGF_, promotes a more rapid development of the networks and leads to increasingly complex vasculatures, an effect that persists up to a saturation level of *C*_VEGF_ ≈ 25 ng/ml VEGF. This is consistent with previous reports,^44,61,62,75–78^ which focused on various biological systems, including *ex-vivo* aortic ring models,^77,78^ EC spheroids,^44^ and EC-coated microcarrier beads^61^ as well as vasculature on chip models,^62^ in which VEGF concentrations ranging from app. 30 to 50 ng/ml were observed to induce the fastest growth of vascular networks and promote bifurcations. Interestingly, similar to our study, Knezevic *et al.*^75^ observed a saturation of vascular network formation at *C*_VEGF_ ≈ 25 ng/ml VEGF without a significant increase when cultures were treated with *C*_VEGF_ = 50 ng/ml. Heiss *et al.*^44^ reported an increase in the length of HUVEC sprouts for VEGF concentrations only up to *C*_VEGF_ = 32 ng/ml, followed by the lack of further increase at *C*_VEGF_ = 64 ng/ml. Moreover, it seems that the range of VEGF concentrations corresponding to the saturation of growth depends on the type of hydrogel. Indeed, the microvasculatures cultured in fibrin matrices tend to saturate at slightly lower VEGF concentrations^44,75^ as compared to the collagen matrices.^34,44,76^ However, the results of several other studies^30,79^ provide contradicting experimental evidence, namely, a non-monotonic response of the EC growth dynamics to *C*_VEGF_. For example, Nakatsu and colleagues^30^ reported a sharp peak in the number of endothelial sprouts (most likely primary branches, but this has not been specified by the authors) as a function of *C*_VEGF_ in cultures where HUVEC-coated beads were treated with 2.5 ng/ml of VEGF, while in assays with *C*_VEGF_ < 1 ng/ml or >10 ng/ml, the authors observed the formation of approximately one sprout per bead after 7 days of culture. Exposing EC-coated beads to concentrations of VEGF higher than 2.5 ng/ml did not lead to the formation of additional sprouts but resulted in a gradual increase in the sprout width. Part of the confusion may lie in the use of different sources of the HUVEC cells, e.g., different donors and isolation times.^44^ Another factor is the concentration of serum in the culture media, which could partially account for the observed morphometric differences.^80^ In our study, we used commercial HUVEC cells collected from 50 donors and cultured them in low serum medium, while Nakatsu *et al.* used freshly isolated HUVEC cells from a single donor and cultured the cells in high serum medium. The increased serum concentration was previously argued to inhibit the endothelial tube formation.^80^

Regarding the dependence of the complexity of the networks on VEGF, we observe that increasing the concentration of VEGF leads to a larger number of primary branches as well as an increase in the average number of branch generations *G*. In particular, in the cultures treated with the highest VEGF concentrations (*C*_VEGF_ = 25 and 50 ng/ml), the period of formation of primary branches is followed by the phase of intense branching, resulting in network densification, a process that we do not observe to occur in cultures deprived of exogenous VEGF or in those treated with low VEGF concentrations (<2.5 ng/ml). Based on our results, we propose that the emergence of more complex morphologies is most likely related to the earlier onset of growth of the network and to the simultaneous emergence of multiple primary sprouts rather than with the faster linear rate of growth of the sprouts. In fact, we find that the maximal speed of the sprout tips is only weakly dependent on the VEGF concentration [Fig. 3(b-i)].

### Exponential distributions of segment length

The origin of the branched morphology of vascular networks *in vivo* can be related to the proliferative activity of equipotent sprout tips that stochastically bifurcate and randomly explore their environment, competing for space.^68^ In the present study, we find that—for relatively high VEGF concentrations—the distribution of lengths of the vascular segments between the bifurcation points, formed by ECs sprouting from EC-coated beads *in vitro*, decays exponentially. This in turn suggests a purely stochastic branching process with a constant bifurcation probability per unit sprout length.

Stochastically branching structures are ubiquitous in nature and can be observed both at the level of multicellular organs, such as lungs,^81^ kidneys,^82^ the pancreas,^83^ mammary glands,^84^ or vascular systems,^85^ as well as at the level of single cells such as neurons^69,86^ or tracheal cells.^87^ The phenomenon is universal across species^88^ ranging from prokaryotic organisms^89,90^ through invertebrates^87^ to vertebrates.^69,81^ Based on the observations, the growth process could be likely modeled by a branching random walk.^68^ However, some of the features of the growing vascular networks are not well reproduced by such models. In particular, in the branching random walks, the growing sprout constantly changes its orientation and, as a result, relatively quickly “forgets” its original growth direction. The growth of our vascular networks, although also random to some extent, is nevertheless on the average directed outwards, away from the initial bead, which can be likely related to the direction of local VEGF- and/or nutrient concentration gradient. The directional growth translates into the linear (or even faster than linear, i.e., superlinear) dependence of the network size on time [see Figs. 3(B-b) and 3(B-f)] shortly after the onset of growth (days 4–8), which is different from the clusters grown in random walk models, such as BARW,^68–70^ the size of which scales sublinearly with time. Random walk models can be adjusted to exhibit radial spreading^69^ by the introduction of an effective radial force, acting on the growing tips. However, it seems more logical to assume that the growth of tips is guided by the VEGF concentration gradients, which produce a deterministic outward motion on top of stochastic, noise-driven effects. In contrast to the radial force model of Ucar *et al.*,^69^ such gradients themselves evolve as the sprouts advance into the matrix and consume VEGF while also locally competing for the VEGF flux. This makes the vascular growth problem similar to the so-called Laplacian growth models^91^ in which the structure grows in the direction of the gradient of the diffusive field.

### Bifurcation angles

In fact, in our experiments, we observe that the average bifurcation angle is typically within the range of 65°–70°, which is close to the theoretical value of the universal branching angle in the Laplacian growth problems, which is equal to 2π/5, i.e., 72°, at least if the growth takes place in a geometry confined to a plane.^71–74^ Noteworthily, similar values of the branching angles were also reported, e.g., in the quasi-planar retina vasculature.^92^ This suggests that diffusive growth might play an important role in the formation of transport networks. This is further confirmed by comparing the characteristic timescales of VEGF diffusion in the system, $\tau_{D}=d_{\text{well}}^{2}/D$, with the characteristic time of an uptake of VEGF by the endothelial cells, $\tau_{R}=1/$ k. In the above, $D\approx 3\times 10^{-7}$ cm^2^/s is the diffusion constant of VEGF, $d_{\text{well}}=$ 1.6 cm is the size of the well, and k≈ 10^−5^/s is the VEGF uptake rate by the endothelial cells.^93^ The ratio of these timescales, known as the Damköhler number, Da $=kd_{\text{well}}^{2}/D\approx$ 85 ≫ 1, i.e., the system is strongly diffusion-limited and the growth is expected to follow the local VEGF gradients. Accordingly, one is tempted to use analogies between growing vascular systems and evolving non-biological networks, such as river networks or crack patterns,^72,94^ to better understand the dynamics of vascular networks as well as other types of living cellular networks, a consideration that we leave for future studies.

### Ambiguities in morphological analysis

**FIG. 6.**
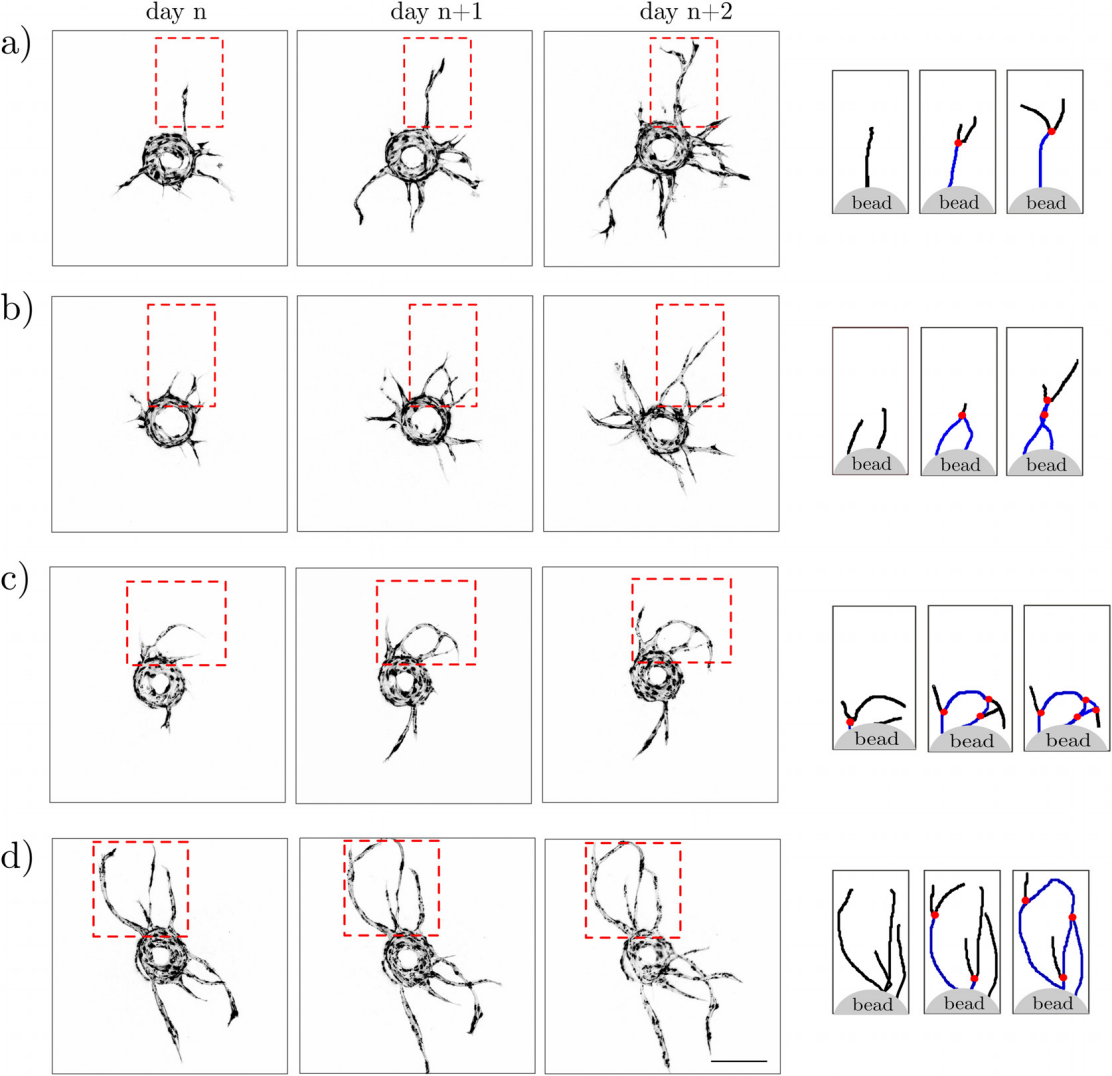
Various scenarios of segment/node formation during development of an EC bead-sprouting network. Confocal images from three consecutive days of culture and schematic representations of the extracted skeletons of the network with indicated nodes (red dots), tip segments (black lines), and bifurcating segments (blue lines) as would be detected by the algorithm. One can distinguish three different scenarios of segment/node formation: (a) bifurcation of a mother branch into two daughter branches, (b) anastomosis (fusion) of two independent branches; here, anastomosis is followed by another bifurcation, i.e., the formation of an X-shaped structure, and [(c) and (d)] the formation of vascular loops caused by sequences of bifurcation and anastomosis events, also including tip-to-tip anastomosis. The scale bar is 250 *μ*m.

Nevertheless, one should also be aware that biological networks are often formed via morphological processes other than branching, yet also resulting in the apparently “branched” structure.^69,89^ In our case, for example, we observe that the nodes of the network are formed not only via bifurcation of the sprout tips [Fig. 6(a)] but may also form via (i) sprout fusion (anastomosis), (ii) partial fusion followed by splitting, resulting in the formation of an X-like structure [Fig. 6(b)], or (iii) formation of a vascular loop via tip-to-tip anastomosis [Figs. 6(c) and 6(d)]. The relevance of the above-mentioned different morphogenetic scenarios remains an open issue, and we leave it for future investigations. Noteworthily, the classification of the nodes of the network with respect to the corresponding scenario of formation would require the development of new sprout-tracking algorithms. The fast development of machine-learning (ML) based tools^95,96^ promises possible applications of ML also in this direction.

### Image analysis software

At the end, we discuss the new functionalities of our custom image analysis toolset developed for the purpose of this work. Biological image analysis has spurred the development of numerous tools to aid the interpretation and quantification of experiments, including open-source platforms, such as ImageJ,^97^ extended by FIJI,^98^ CellProfiler,^99^ Vaa3D,^100^ Icy,^101^ and others, as well as commercial tools (such as Imaris, Amira, or Volocity). Particularly popular platforms for angiogenetic analysis are Sprout Analysis plugin to ImageJ,^102^ Matlab-based AngioQuant for *in vitro* assays,^103^ or AngioIQ,^104^ which uses a graphic interface, as well as SproutAngio tool written in Python.^105^ Carpentier *et al.* developed Angiogenesis Analyzer^108^ and recently applied it to fibrin-based assays.^41^ There is also a commercially available IKOSA AI tool,^106^ which exploits machine-learning algorithms. These tools offer efficient ways to segment and skeletonize images of angiogenic networks and provide insights into the length and area statistics by resolving junctions and branches.

We have performed a basic comparison between our software and the three chosen software: Sprout Analyzer,^102^ Sprout Angio,^105^ and IKOSA AI.^106^ The details of the comparison are given in the supplementary material, and here, we provide a short summary. First, we note that our software is tailored to the specific requirement of our work, which includes parallel processing of multiple images of different beads acquired at different time-points and their topological analysis. However, we do not assert that it is overall superior to the other free and commercial solutions available on the market, which often focus on different types of tasks (analysis of multi-channel data, 3D analysis, constant improvement of performance via machine learning).

Notably, our software exhibits exceptional speed, likely ranking as one of the fastest among the compared solutions (see supplementary material, Chapters 2.1, 3.1, and 4.1). It is written in a contemporary programming language, characterized by ease of acquisition and supported by a vibrant community, facilitating its adoption by a wider audience. Our software introduces novel metrics, offering comprehensive control over graph manipulation and providing extensive debugging information. It is suited to the format and character of the measured data and tailored to the detection of primary branches and outer branches, necessary for the analysis of bifurcations and branching angles, which the other tested tools are lacking.

Moreover, we have incorporated tools for clean data storage and statistical analysis, which have not been developed to the similar extent in the compared software. A substantial portion of the codebase is subjected to rigorous unit testing, ensuring its robustness. Furthermore, our software separates the implementation layer from the Application Programming Interface (API), a feature not readily apparent in other solutions, e.g., IKOSA AI, where API access is restricted, while in solutions such as SproutAngio and Sprout Analyzer, the software is provided in the form of scripts. In the compared software, the users are constrained to perform the analysis through the graphical user interface (GUI), which is time-consuming for larger datasets.

In summary, we consider our software to be a valuable tool, underpinned by a strong foundation, well-suited for the specific challenges posed by the time-resolved analysis of the bead-sprouting angiogenesis. While certain limitations persist, such as those related to the working with 2D projections instead of the full 3D data and with rare ambiguities during processing, we continue to work toward their refinement and resolution. Last but not least, we note that, with novel tools exploiting the advantages of deep learning coming into play,^12^ including the already available IKOSA AI software, we expect that AI will play an increasingly important role in the angiogenesis image analysis.

## SUMMARY AND CONCLUSIONS

In this work, we have reported high-throughput fibrin-gel angiogenesis bead-assays aimed at long-term tracking of the evolution of sprouting vascular networks, in particular, focusing on a variety of statistical morphological/topological characteristics of the networks. To this end, we have developed Python-based software for the morphometric analysis of vascular networks. We used this tool to demonstrate how the dynamics of the growth of HUVEC sprouts depends on the distribution of fibroblasts in the surrounding hydrogel matrix and on the concentration of VEGF in the medium.

Overall, we have demonstrated the possibility of extracting detailed statistical–topological features of bead-sprouting microvascular networks at high throughput. Our findings can be of practical relevance in the development of angiogenesis drug testing assays as well as in tissue engineering. In the former case, our methodology and software could greatly enhance the outcomes of high throughput screening studies, such as, e.g., the one performed by Heiss *et al.*,^44^ who evaluated the pro- and antiangiogenic capacity of over 800 chemical compounds using sprouting EC-spheroids. The sprouting intensity was assessed manually by categorizing the compounds into four groups based on the microscopic observations. Our software could greatly enhance such kind of assays via providing a more robust and significantly expedited workflow. In addition, our software would also deliver a wider range of morphometric measures and provide quantitative insights into the details of drug effects on the emerging vascular phenotype. Noteworthily, the screening of large libraries is one of the first and most critical steps toward identification of potential drug candidates. Thus, having a robust, hands-off system capable of fast quantitative analysis remains of a great importance in a variety of preclinical R&D pipelines.

Finally, in terms of tissue engineering, our results could be used to define the optimal conditions for efficient vascularization in the biofabrication strategies based on the use of endothelial seeds^19,20,22,24,25,27,29,107^ . In such strategies, multiple seeds are initially dispersed in the external ECM to eventually interconnect and form a fully percolated mesoscale network. Whereas the approach has been initially exploited with random-sized microvessel fragments as the seeds,^107^ the fabrication of seeds of more controlled size and shape, such as EC-encapsulating hydrogel beads made of fibrin, agarose,^22,24,108^ or polyethylene glycol (PEG) derivatives,^19^ has also been demonstrated. Here, we have focused on a single cell-coated sprouting seed, but our systematic approach could be further extended to the case of modular microvasculatures. Such future studies could potentially provide for better understanding of how the external cues affect vascularization in biomaterials with embedded endothelial seeds and help to optimize tissue repair strategies, e.g., via proper design of the prevascularized wound dressings.^10,109^

## METHODS

### Cell culture

GFP-HUVECs (Angio-Proteomie, Boston, MA; catalog no. cAP-0001GFP) were cultured in endothelial cell growth medium 2 (EGM-2) medium supplemented with EGM-2 bulletkit (Lonza, Basel, Switzerland; catalog no. CC-3156 and CC-4176) and were used at passages 3 through 5. NHDF (Promocell, Heidelberg, Germany; catalog no. C-12302) were cultured in Dulbecco's modified Eagle medium (DMEM) (Thermo Fisher Scientific, Waltham, MA, USA; catalog no. 10566016) supplemented with 10% fetal bovine serum (FBS), 4.5 g/l of glucose, Glutamax, and 1% penicillin–streptomycin. NHDFs were used between passages 2 and 7. All cells were cultured in 5% CO_2_ at a 37 °C humidified atmosphere, and media were replaced every 2 days.

### Fibrin bead-sprouting assay

Coating of the beads with EC was performed as described previously^110^ with small modifications. Briefly, HUVECs-GFP were mixed with 265 *μ*m diameter monodispersed polystyrene superparamagnetic microcarrier beads (microParticles GmbH, Berlin, Germany; catalog no. PS-MAG-AR111) at a concentration of approximately ∼500 cells per bead in a small volume of warm EGM-2 medium and placed in the incubator for 4 h at 37 °C and 5% CO_2_, gently shaking the tube every 20 min. After 4 h, beads were transferred to a culture flask with fresh EGM-2 medium and placed overnight in the incubator at 37° C and 5% CO_2_. The following day, beads were gently washed with EGM-2 medium, resuspended in freshly prepared 2.5 mg/ml fibrinogen solution (Sigma-Aldrich, St. Louis, MO, USA; catalog no. 341573), mixed with 0.625 units of thrombin (Sigma-Aldrich, St. Louis, MO, USA; catalog no. T4648), and seeded one bead per well in a 24-well plate. After pouring the fibrin hydrogel with one EC-coated bead into each well, we inserted a sheet of paper with marked spots underneath the well-plate for better visualization of the beads and also for use as a template for precise bead positioning. We used titanium fine tip tweezers to position the beads above the marked spots, centrally in the wells. Fibrin/bead solution was allowed to clot for 5 min at room temperature and then at 37° C and 5% CO_2_ for 30 min. 1 ml of EGM-2 medium was added to each well. NHDFs at a concentration of 25 000 cell/well were either layered at the top of the clot or added to the fibrin/bead solution before seeding the beads. The medium was changed every day. Human recombinant VEGF-165 (Stemcell technologies; Saint Egrève, France; catalog no.78073) was used at the indicated concentrations. In experiments where EC-coated beads were treated with exogenous VEGF-165, we used EGM-2 medium deprived of VEGF solution provided by the manufacturer. This was done to reduce the impact of other VEGF isoforms that could be present in the commercial solution and to avoid any additional VEGF supplementation in the medium that could affect the final concentration of VEGF in the experimental setup.

### Immunofluorescence staining

Fibrin blocks with NHDF cells were fixed with 4% paraformaldehyde (PFA) and blocked with a blocking buffer (2% bovine serum albumin [BSA], 2% normal goat serum, and 0.5% Triton X-100 in PBS). Actin-488 conjugated antibody (Sigma-Aldrich, St. Louis, MO, USA; catalog no. ABT1485-AF488) was applied in a blocking buffer.

### Image acquisition and processing

EC-coated beads were imaged every 24 h for 14 consecutive days using a Nikon A1 confocal microscope (Nikon Instruments, Inc, Melville, NY, USA) equipped with a PLAN APO 10×/0.45 objective. Images were collected using NIS-Elements Advanced Research software (Nikon Instruments, Inc, Melville, NY, USA) in the nd2 format, which are 16-bit single-channel images with respective metadata. Each experiment was recorded as a set of successive frames, where a single frame had several slices in the z-direction. The resolution of images is 1.25 *μ*m/pixel. The images were first max-pooled on the z-directional slices (taking the maximum intensity value across the stack) and treated with a Gaussian blur with a kernel of size 11 pixels. To distinguish cells from the background, segmentation was performed with the threshold based on the average intensity multiplied by 1.17. A filling algorithm was used to eliminate holes with perimeter smaller than 200 pixels (250 *μ*m), and the largest connected component was taken. The centrally located polystyrene bead “mask” was then detected by a top-hat transform algorithm. To focus the analysis on the geometrical characteristics of the growing sprouts, the central bead was removed using the previously detected mask. The processed images were subsequently skeletonized using an algorithm from the scikit-image package. Connectivity of the resulting skeletonized network was then determined by identifying branching points (junctions) and segment tips. Finally, tip segments shorter than 50 pixels (62.5 *μ*m) were pruned, except for those connected to the mask (base of a sprout). The exact values of the applied parameters (blur kernel size, minimal hole size, pruning length, arm length in angle measurement, etc.) used in the image processing have been chosen to maximize the agreement with the results of the direct manual image segmentation performed by two independent researchers. After calibration on a representative data subset, high-throughput measurements were performed on wider data sets. In our software, the parameters can be tuned to fit the conditions of the experiment, which provides a high level of flexibility for various applications.

### Geometrical and topological characteristics of the sprouting network

Based on the segmented pictures, skeleton, and mask, we computed the geometrical characteristics of the sprouting network (*A*_c_, *A*, *L*, *r*_max_, *N*_pb_, *N*_tip_, *λ*, *G*). Moreover, for each experiment, we detected the bifurcations points, that is, the nodes in the graph with three outgoing segments, and measured three angles associated with each bifurcation point. The angles were measured between straight lines connecting the bifurcation point and a selected point on each of the outgoing branches. Because of the finite thickness of the sprouts, the process of skeletonization resulted in branches being slightly curved toward the branching point. Accordingly, if one selected the points for the angle measurement very close to the bifurcation point, then the computed bifurcation angle would appear larger than in the case with the points selected at a larger distance from the bifurcation. The importance of choosing the right length scale for the measurements of bifurcation angles was highlighted before, e.g., in the context of geometric characteristics of river networks.^111^ We decided to select the points at 60 pixels (approximately 75 *μ*m) from the bifurcation point (calculated along the sprout) on each branch as in this case we obtained the results that best matched the manual measurements (see supplementary material, Fig. 5).

It is important to note that out of the three angles adjacent to a bifurcation point, one needs to select the actual bifurcation angle, that is, the angle between the two *daughter* branches. In general, based on the manual segmentation of several images, we found that the bifurcation angles are typically between 30° and 120°. Therefore, in the algorithm, we took the smallest of the three angles as the supposed bifurcation angle. We note that this method has a limitation in the sense that it cannot yield bifurcation angles larger than 120°. Also, the smallest angle at a selected node may as well be associated with anastomosis of the vessels rather than bifurcation. In order to exclude the angles corresponding to anastomosis events, we checked whether the bisector of the selected smallest angle pointed inwards or outwards with respect to the polystyrene bead (the center of the mask). Only the cases with the bisector pointing outwards (i.e., with the maximum angle of 90° between the bisector and the vector connecting the center of the bead and the node) were classified as bifurcations.

Finally, the fitting of the exponentially decaying function to the PDF of branch lengths *P*(*l*) was done using the semi-logarithmic scale. That is, we fitted a linear function with a negative slope to the dataset ln(*P*(*l*)) vs *l*. To this end, we used a linear least squares regression implemented in library SciPy (function *linregress*). The fitting of a sigmoid function from Eq. (1) to the experimental data *L*(*t*) [Fig. 3(c-a)] and *A*(*t*) [supplementary material, Fig. 4(a)] was conducted using the non-linear least squares method (function *curve_fit* from SciPy).

### Statistical analysis

Directly measured quantitative data are expressed as the mean ± SEM. The statistical methods (two-tailed and unpaired *t* tests, one-way analysis of variance followed by Tukey's post hoc test, and two-way analysis of variance followed by Bonferroni's post hoc test, Shapiro–Wilk test, U Mann–Whitney test) and *p*-values are defined in the figure legends or in supplementary material Table 1 (S1 Table). The quantification of the manually measured parameters (bifurcation angles, tip, and bifurcation segments) was performed in a blinded manner and confirmed by two independent researchers. The statistical analyses were performed using GraphPad Prism 5 and Microsoft Excel software, and the scipy stats Python module.

Evaluation of the logistic fit performance was measured by MAPE (mean absolute percentage error) and WMAPE (weighted mean absolute percentage error). The MAPE metric is defined as

$$\mathrm{MAPE}=\frac{1}{n}\sum_{i=1}^{n}\left|\frac{x_{i}-x_{p}}{x_{i}}\right|,$$

where *n* is the number of points used for fitting, *x_i_* is the actual fitting value, and *x_p_* is the corresponding predicted value by the model. It has drawbacks in the case of very small values, which can cause infinite error. Hence, we also use the second metric WMAPE, which is weighted by the sum of all contributions (fitting points)

$$\mathrm{WMAPE}=\frac{\sum_{i=1}^{n}\left|x_{i}-x_{p}\right|}{\sum_{i=1}^{n}\left|x_{i}\right|}.$$

Comparison of fits for segment distribution is done using the *n*-sigma test defined as

$$x_{1}-x_{2}\leq n\sigma =n\sqrt{u_{1}^{2}+u_{2}^{2}},$$

where *n* is an integer, *x*_1_ is value for the first fit, *x*_2_ for the second fit, and *u*_1_, *u*_2_ are the corresponding uncertainties. If the condition is not met (“false”), one cannot assume that observations came from the same random process, i.e., there is a statistical difference between the measured values *x*_1_, *x*_2_. Otherwise (“true”), there is no statistical difference.

The results of the statistical analysis are available in the following sheets of S1 Table:

Figure 2(b): two-way ANOVA with the Bonferroni post hoc test to detect statistical differences between the NHDF monolayer and NHDF intermixed.

Figure 2(c): Shapiro–Wilk and Mann–Whitney U tests validate differences in bifurcation angle distributions between the NHDF monolayer and NHDF intermixed.

Figure 3(b): two-way ANOVA with the Bonferroni post hoc test detects statistical differences among various VEGF concentrations.

Figure 3(c) and supplementary material, Fig. 4: Sigmoid parameters derived from fitting, including the onset and the maximum growth speed for the area and the total length, with uncertainties, MAPE, and WMAPE metrics.

Figure 4: one-way ANOVA with Tukey's post hoc test to assess similarities in the bifurcation angle distributions among various VEGF concentrations.

Figure 5, supplementary material, Figs. 6 and 7: 3-sigma tests comparing characteristic lengths at 25 and 50 ng/ml for various observers (algorithm and researchers). The “Comparison” has the value “True” if the 3-sigma test is satisfied and “False” otherwise. The second table compares the characteristic length values between observers. Additionally, a comprehensive table is included that compares the tip and the bifurcation characteristic lengths for low VEGF concentrations using both 2-sigma and 3-sigma tests. The test result is indicated by True or False, depending on whether the inequality is satisfied or not.

Supplementary material 5: Mann–Whitney U tests comparing the bifurcation angle distributions for various arm lengths with the researchers' results.

## SUPPLEMENTARY MATERIAL

See the supplementary material for supplementary Figs. 1–7, which show (i) additional confocal images of GFP-tagged HUVEC-coated beads cultured at different conditions, (ii) graphs characterizing the dynamics of EC sprouting at various VEGF concentrations, (iii) details of the branching angle calculation methodology and its validation, (iv) validation of the measurements of the segment length distribution at different VEGF concentrations, and (v) distribution of segment lengths for lower VEGF concentration.
The supplementary material also includes the supplementary information file presenting a comparative study of bead-sprouting morphology analysis tools including our software and three common competitors, and two tables (i) S1 Table showing the detailed statistical analysis for Figs. 2–5 and supplementary material, Figs. 4, 6, and 7 and (ii) S1 Data including all numerical values underlying the graphs in the manuscript.

## Data Availability

The data that support the findings of this study are available within the article and its supplementary material.
